# Clara Cell Protein Expression in Mechanically Ventilated Term and Preterm Infants with Respiratory Distress Syndrome and at Risk of Bronchopulmonary Dysplasia: A Pilot Study

**DOI:** 10.1155/2017/8074678

**Published:** 2017-04-11

**Authors:** José Guzmán-Bárcenas, Antonio Calderón-Moore, Héctor Baptista-González, Claudine Irles

**Affiliations:** ^1^Department of Perinatal Hematology, Instituto Nacional de Perinatología Isidro Espinoza de los Reyes, Montes Urales 800, 11000 Lomas de Virreyes, CDMX, Mexico; ^2^Neonatal Intensive Care Unit, Hospital Infantil de México, Dr. Márquez No. 162, Cuauhtémoc, 06720 Doctores, CDMX, Mexico; ^3^Department of Physiology and Cellular Development, Instituto Nacional de Perinatología Isidro Espinoza de los Reyes, Montes Urales 800, 11000 Lomas de Virreyes, CDMX, Mexico

## Abstract

The aim of this pilot study was to determine Clara cell protein (CC16) concentration in bronchoalveolar lavages (BAL) fluid from full-term and preterm (<37 weeks' gestational age) neonates requiring respiratory support, having symptoms of neonatal respiratory distress syndrome, and at risk of bronchopulmonary dysplasia (BPD). We hypothesized that CC16 may be predictive of BPD diagnosis regardless of gestational age. BAL fluid CC16 was measured by ELISA at birth and at day 7 of life. Both groups that developed BPD showed significantly decreased BAL fluid CC16 levels compared to those infants that did not develop the disease. CC16 positively correlated with diagnosis of BPD and negatively with the severity of the disease. These results suggest that BAL fluid CC16 levels may have a diagnostic value at day 7 for BPD in both term and preterm infants. This study demonstrates the potential utility of BAL fluid CC16 levels as a biomarker for BPD in term infants.

## 1. Introduction

Bronchopulmonary dysplasia (BPD) also called chronic lung disease (CLD) of infancy is a neonatal-pediatric disease associated with lung injury in preterm infants or anomalous lung development in full-term neonates [1–3] and is usually associated with neonatal respiratory distress syndrome (RDS). BPD has been linked with long term pulmonary morbidity and function [4] and neurodevelopmental sequelae [5] during infancy. BPD is physiologically defined by NIH Workshops [6–8] as a requirement for supplemental oxygen for >28 days and severity of the disease is graded according to gestational age (necessity for mechanical ventilation occurs in severe BPD). However, it requires a period of time before BPD diagnosis and severity is determined. Therefore, biomarkers that allow early diagnosing and grading severity of BPD should be very useful to early predict this disease.

Clara cell secretory protein of 10–16 kDa (CC16), also known as CC10, Club cell protein, CCSP, or uteroglobin [9, 10], a member of the secretoglobin family [11], is the most abundant protein in bronchoalveolar lavage (BAL) fluid or tracheal aspirates (TA) from neonates [12, 13]. CC16 plays anti-inflammatory, immunomodulatory, and airway repairing roles [14–16]. Available studies have identified CC16 as a potential marker for BPD in preterm infants (reviewed by [17, 18]) but with contradictory results with either decreased [19, 20] or increased [21] cord blood CC16 levels or lower TA CC16 levels [13]. However, CC16 studies in full-term infants at risk of developing BPD are still scarce. Here we tested the hypothesis that CC16 may be predictive of BPD diagnosis regardless of gestational age. Therefore, the aim of this exploratory pilot study was to determine CC16 levels in BAL fluid from term and preterm infants with respiratory failure, mechanically ventilated that subsequently developed BPD.

## 2. Materials and Methods

The Institutional responsible Ethical Committee and Research Committee of the National Institute of Perinatology* Isidro Espinoza de los Reyes* approved the study (#212250-19071) and signed informed consent was obtained. The study was conducted according to the Declaration of Helsinki (1964) ethic principles for human participants.

### 2.1. Clinical Characteristics

Ten newborns were included in the study: term (>37 weeks' gestation) and preterm (28–36 weeks' gestation) neonates who required endotracheal intubation for mechanical ventilation for more than 7 days and diagnosis of failure of ventilation treatment (3), with signs and symptoms of neonatal respiratory distress syndrome (RDS), in whom bronchopulmonary dysplasia (BPD) was diagnosed subsequently (*n* = 5) or not (*n* = 5). Clinical data was collected from medical records such as gestational age, birth weight, gender, Apgar score, prenatal steroids, and surfactant treatment. One infant had prophylactic surfactant, one neonate received rescue surfactant, and, finally, one infant had one more dose of rescue surfactant. Exclusion criteria were infants with chromosomal abnormalities, congenital lung and heart diseases, and malformations. The clinical characteristics of the study population are depicted in Table 1.

### 2.2. Clinical Characteristics of Term Neonates

Only term neonates presented the following characteristics: septic shock (two neonates) and transitory tachypnea of the newborn (three neonates) with two of the latter newborns also developing transitory pulmonary hypertension. Therefore, neonatal mechanical ventilation was secondary to the initial diagnosis.

BPD diagnosis and severity were defined according to the National Institute of Child Health and Human Development (NICHD) and National Heart, Lung, and Blood Institute (NHLBI) Workshops [6–8]: Grade 1 BPD (mild): supplemental oxygen for at least 28 days and on room air at 36 weeks' PMA/discharge (for infants < 32 weeks at birth) or at 56 days/discharge (for infants > 32 weeks at birth); Grade 2 BPD (moderate): supplemental oxygen for at least 28 days and receiving supplemental effective oxygen < 30% at 36 weeks/discharge (for infants < 32 weeks at birth) or 56 days/discharge (for infants > 32 weeks at birth); Grade 3 BPD (severe): supplemental oxygen for at least 28 days and receiving supplemental effective oxygen > 30% or on nasal CPAP or mechanical ventilation at 36 weeks/discharge (for infants < 32 weeks at birth) or 56 days/discharge (for infants > 32 weeks at birth) [8].

### 2.3. Collection of Samples and CC16 Determination

Bronchoalveolar lavages (BAL) fluid samples were collected only when indicated clinically (i.e., endotracheal intubation), at day 1 (birth) and day 7 (1 week). A nonbronchoscopic method was used to obtain tracheal aspirate. Briefly, after tracheal intubation, a fixed volume of saline solution (0.5 mL) was applied and immediately aspirated (the recovered volume was 0.3 mL). The fluid was kept at all times at 4°C, under ice, and transported to the laboratory within 30 min after its collection. Samples were immediately centrifuged for 10 min at 3500 rpm/4°C and the supernatant was frozen at −70°C. Total protein concentration in BAL samples was determined by Bradford assay (Bio-Rad, California, USA). CC16 protein concentration was assayed in BAL fluid in duplicate by a commercial CC16 ELISA kit (RD191022200, Biovendor, Brno, Czech Republic) with an intra-assay coefficients of variation (CV) of 3.4% and interassay CV of 4.7%.

### 2.4. Statistical Analysis

Results are expressed as mean ± SEM, [range], or number of individuals as appropriate. Statistical differences were assessed by Mann–Whitney *U*, two-tailed test [22]. Correlations were evaluated using Pearson test. For all analysis, values of ^*∗*^*p* < 0.05 and ^*∗∗*^*p* < 0.01 were considered to be statistically significant. Analysis was performed by SPSS 16.0 software (IBM, Armonk, NY, USA).

## 3. Results

In this pilot study, the objective was to determine Clara cell protein (CC16) concentration in BAL fluid from term and preterm infants with respiratory failure in whom bronchopulmonary dysplasia (BPD) was diagnosed subsequently. All neonates required ventilatory support for more than 7 days. The peak inspiratory pressure exceeded 17 cm H_2_O in six infants. BPD was diagnosed in five infants from which three were term and two were preterm neonates. One neonate presented Grade 1 BPD (one preterm neonate), three neonates Grade 2 BPD (two term and one preterm neonates), and one neonate Grade 3 BPD (one term neonate). The clinical characteristics are depicted in Table 1. Five neonates presented an appropriate for gestational age weight while the other five had a weight of less than 2500 g. Intrauterine growth restriction (IUGR) was diagnosed in five infants. Maternal conditions were gestational diabetes mellitus in one neonate, chorioamnionitis in one infant, and preeclampsia in two neonates. Prophylactic surfactant was administered in one of the infants.

BAL fluid CC-10 levels were found significantly diminished in neonates that developed BPD compared to those that did not develop the disease (0.43 ng/mL ± 0.13 versus 2.76 ng/mL ± 0.57, *p* = 0.0079^*∗∗*^ in day 1 and 0.29 ng/mL ± 0.06 versus 1.07 ng/mL ± 0.29, *p* = 0.0317^*∗*^ in day 7; Table 2) with no statistical differences between term and preterm infants. BAL fluid CC-10 concentrations obtained at day 7 were found to be decreased in comparison with day 1 (*p* = 0.0159^*∗*^ and *p* = 0.5476 without or with BPD, resp.; Table 2).

There was a significant correlation between both CC16 determinations in BAL with BPD diagnosis (*r* = 818^*∗∗*^, *p* = 0.004 and *r* = 683^*∗*^, *p* = 0.029, two tails for days 1 and 7, resp.) and severity of the disease (*r* = −0.769^*∗∗*^, *p* = 0.009 and *r* = −0.680^*∗*^, *p* = 0.031, two tails for days 1 and 7, resp.). Furthermore, CC16 levels at day 1 also correlated with maximal weight loss at day 7 (15% of total weight) (*r* = −0.695, ^*∗*^*p* = 0.026, two tails). IUGR, prophylactic, and rescue surfactant did not correlate significantly with any variable.

These results suggest that BAL fluid CC16 levels have a diagnostic value on day 7 for BPD in both term and preterm infants.

## 4. Discussion

Both term and preterm neonates that developed BPD had lower BAL fluid CC16 levels in comparison with neonates that did not develop the disease. This pilot study is in agreement with previous studies for preterm infants [14, 23] but provides further evidence for term infants. Furthermore, CC16 levels at birth were found to positively correlate with BPD diagnosis and negatively with severity. These results suggest that BAL fluid CC16 concentration will be able to help to early discriminate between infants with none, mild, moderate, and severe BPD, independently of gestational age.

Interestingly, CC16 levels also correlated with optimal weight gain and this result may point to the importance of optimal nutrition for repairing lung injury and increasing the energy needs caused by BPD.

The physiopathological mechanisms of BPD in term and preterm infants are probably different. Preterm birth is associated with lung immaturity, oxygen toxicity (oxygen free radical exposure), and mechanical ventilation (reviewed by [24, 25]). Further research is needed in order to elucidate the mechanisms of BPD in term neonates which is probably distinct in preterm neonates.

It has been shown in a mouse model that CC16 administration increases epithelial proliferation, protects against oxidative stress [26], and is suggested to be linked to a population of Clara cells, producing CC16 protein, with a bone marrow (BMC) phenotype which would be implicated in lung repair by reducing pulmonary inflammation and promoting airway regeneration [27]. In our neonates, it is possible that the low levels of CC16 protein related to BPD severity are due to a diminished number of Clara cells affecting lung recovery.

### 4.1. Limitations of the Study

We acknowledge that the results and analysis are limited by the very small sample size; however, we developed this pilot study with the intention to incorporate a wider study in our own center and other centers based on the obtained results. This exploratory work will contribute to the development of a subsequent study by the information obtained of BAL CC16 concentration in BPD.

## 5. Conclusions

BAL fluid CC16 levels were found to be decreased in both term and preterm ventilated neonates who subsequently developed BPD. CC16 levels at day 7 may be useful to diagnose the disease. This study demonstrates the potential utility of BAL fluid CC16 levels as a biomarker for BPD in term infants.

## Figures and Tables

**Table 1:** Clinical characteristics in neonates without or with BPD^*∗*^.

	No BPD (*n* = 5)	BPD (*n* = 5)
Gender Female, male	3, 2	2, 3
Gestational age (weeks)	34.2 ± 2.2 [28–39]	34.8 ± 1.8 [29–39]
Preterm < 36 weeks	3	2
Term > 37 weeks	2	3
Birthweight (g)	2120 ± 453 [750–3150]	2266 ± 462 [900–3250]
Apgar score at 1 min	6.2 [5–7]	7
Apgar score at 5 min	7.4 [5–8]	7.8 [7-8]
GDM	0	1
Chorioamnionitis	0	1
Preeclampsia	0	2
IUGR	2	3
Death	0	1
Prenatal steroids	0	1
Prophylactic surfactant	0	1
Atelectasis	3	1

^*∗*^Values are depicted as mean ± SEM, [range], or number of individuals.

GDM: gestational diabetes mellitus; IUGR: intrauterine growth restriction.

**Table 2:** BAL fluid CC16 concentration in ventilated term and preterm neonates from day 1 and day 7.

	GE (weeks)	BAL CC10 (ng/ml)				BPD severity
		No BPD		BPD		
		*Day 1*	*Day 7*	*Day 1*	*Day 7*	
Neonate 1	37	—	—	0.89	0.35	II
Neonate 2	37	—	—	0.36	0.26	II
Neonate 3	38	2.84	0.49	—	—	no
Neonate 4	39	2.13	1.32	—	—	no
Neonate 5	39	—	—	0.097	0.12	III
Neonate 6	28	1.63	1.73	—	—	no
Neonate 7	29	—	—	0.35	0.23	II
Neonate 8	30	4.89	1.53	—	—	no
Neonate 9	32	—	—	0.45	0.47	I
Neonate 10	36	2.33	0.27	—	—	no
Mean		2.76	1.07	0.43	0.29	
SEM		0.57	0.29	0.13	0.06	
